# Signaling Mutations Negate the Favorable Impact of *NPM1* Mutations in Older Patients With Newly Diagnosed Acute Myeloid Leukemia Treated With VEN/HMA

**DOI:** 10.1002/ajh.70419

**Published:** 2026-07-02

**Authors:** Fieke W. Hoff, Joshua F. Zeidner, Geeta Torlapati, Deedra Nicolet, Krzysztof Mrózek, Ying Huang, Alexander Li, Rina Li Welkie, Ronan T. Swords, Elie Traer, Eytan M. Stein, Tara L. Lin, Maria R. Baer, Vu H. Duong, William G. Blum, Martha L. Arellano, Wendy Stock, Olatoyosi Odenike, Rebecca L. Olin, Catherine C. Smith, Gary J. Schiller, Emily K. Curran, Onyee Chan, Christine McMahon, Michael Hochman, Kieran Sahasrabudhe, Charles Foucar, Jesus Gonzalez‐Lugo, Brittany Knick Ragon, Shivani V. Handa, Nyla A. Heerema, Timothy Chen, Molly Martycz, Mona Stefanos, Sonja G. Marcus, Leonard Rosenberg, Brian J. Druker, Ross L. Levine, Amy Burd, Ashley O. Yocum, Uma M. Borate, Alice S. Mims, Ann‐Katrin Eisfeld, John C. Byrd, Yazan F. Madanat

**Affiliations:** ^1^ National Heart, Lung, and Blood Institute National Institutes of Health Bethesda Maryland USA; ^2^ Division of Hematology, Department of Medicine, Lineberger Comprehensive Cancer Center University of North Carolina Chapel Hill North Carolina USA; ^3^ The James Cancer Hospital The Ohio State University Columbus Ohio USA; ^4^ Clara D. Bloomfield Center for Leukemia Outcomes Research The Ohio State University Comprehensive Cancer Center Columbus Ohio USA; ^5^ Department of Internal Medicine University of Texas Southwestern Medical Center Dallas Texas USA; ^6^ Division of Hematology and Medical Oncology, Knight Cancer Institute Oregon Health & Science University Portland Oregon USA; ^7^ Leukemia Service, Department of Medicine, Human Oncology and Pathogenesis Program Memorial Sloan Kettering Cancer Center New York New York USA; ^8^ Division of Hematologic Malignancies University of Kansas Medical Center Kansas City Kansas USA; ^9^ Department of Medicine University of Maryland Greenebaum Comprehensive Cancer Center Baltimore Maryland USA; ^10^ Department of Hematology and Medical Oncology Winship Cancer Institute of Emory University Atlanta Georgia USA; ^11^ Section of Hematology/Oncology University of Chicago Chicago Illinois USA; ^12^ Division of Hematology and Oncology University of California, San Francisco San Francisco California USA; ^13^ Department of Medicine, Hematology/Oncology David Geffen School of Medicine at University of California, Los Angeles Los Angeles California USA; ^14^ Division of Hematology & Oncology University of Cincinnati Cincinnati Ohio USA; ^15^ Malignant Hematology Department H. Lee Moffitt Cancer Center Tampa Florida USA; ^16^ Division of Hematology, Department of Medicine University of Colorado School of Medicine Aurora Colorado USA; ^17^ Division of Hematology University of Wisconsin, Carbone Cancer Center Madison Wisconsin USA; ^18^ Department of Internal Medicine, Division of Hematology and Oncology University of New Mexico Albuquerque New Mexico USA; ^19^ Department of Hematologic Oncology and Blood Disorders, Levine Cancer Institute, Atrium Health Wake Forest University School of Medicine Charlotte North Carolina USA; ^20^ Blood Cancer United Washington DC USA; ^21^ UPMC Hillman Cancer Center University of Pittsburgh Pittsburgh Pennsylvania USA

## Abstract

Frameshift mutations in exon 12 of nucleophosmin 1 (*NPM1* ^mut^) are among the most common mutations in acute myeloid leukemia (AML) and have historically been considered favorable‐risk in the absence of *FLT3*‐ITD. In the European LeukemiaNet (ELN) 2024 risk‐classification for patients treated with hypomethylating agents plus venetoclax (HMA + VEN), *NPM1* ^mut^ is not considered favorable when co‐occurring with signaling gene (SG) mutations (i.e., *FLT3*‐ITD, *NRAS*, *KRAS*). However, due to limited numbers in the original analysis, the prognostic impact of *SG* mutations in *NPM1*‐mutant AML remains unclear. We evaluated the prognostic significance of *NPM1* ^mut^ with and without SG mutations in two independent cohorts of patients ≥ 60 years with ELN 2024 favorable‐ or intermediate‐risk AML treated with HMA + VEN. Cohort 1 included 322 patients treated in the academic setting. *NPM1* ^mut^ (*n* = 61) was associated with a nonsignificantly longer overall survival (OS) compared to *NPM1* wild‐type (*NPM1* ^wt^) (median, 53.05 vs. 17.03 months, *p* = 0.10). In multivariable analysis (MVA), SG mutations were not independently prognostic within the *NPM1* ^mut^ subgroup. Cohort 2 included 816 patients from a real‐world community‐treated cohort. *NPM1* ^mut^ (*n* = 124) had a longer OS compared with *NPM1* ^wt^ (median, 15.3 vs. 14.4 months, *p* = 0.03). In MVA, *NRAS, KRAS*, and *FLT3*‐ITD were independent unfavorable prognostic factors; *NPM1* ^mut^ with, compared to without, SG co‐mutation had a shorter OS (median, 9.4 vs. 31.6 months, *p* = 0.001). These findings suggest SG mutations negate the favorable impact of *NPM1* ^mut^ in older patients treated with HMA + VEN. Prospective clinical trials are needed to investigate the use of combination therapies to improve outcomes in this high‐risk subgroup.

## 1 Introduction

Frameshift mutations in exon 12 of nucleophosmin 1 (*NPM1* ^mut^) are among the most frequent molecular abnormalities in acute myeloid leukemia (AML), occurring in approximately 35% of all patients [1]. In both the World Health Organization (WHO) and the International Consensus Conference (ICC) classifications, *NPM1* ^mut^ AML is recognized as a genetically defined AML subtype [2, 3]. However, due to the frequent co‐occurring mutation profiles and patient characteristics, clinical outcomes remain highly heterogeneous within this molecular subgroup.

Risk stratification based on the revised European LeukemiaNet (ELN) 2022 classification identifies *NPM1* ^mut^ AML as conferring favorable‐risk in the absence of a co‐occurring *FLT3* internal tandem duplication (*FLT3‐*ITD) or adverse risk karyotype [4]. However, ELN 2022 is primarily based on outcomes in younger patients (generally 60 years) receiving intensive chemotherapy (IC), whereas for older patients treated with IC, the prognosis is less clearly favorable [5, 6, 7].

Pooled analysis of 279 patients, including those from an open‐label phase 1b trial [8] and the phase 3 VIALE‐A trial [9, 10], evaluating azacitidine (AZA) plus VEN (AZA + VEN) versus AZA plus placebo in patients who were ineligible for standard induction therapy, assessed clinical outcomes of patients treated with AZA + VEN. Patients with *NPM1* ^mut^ AML had a median OS of 39.0 months in the absence of *FLT3*‐ITD, *KRAS* and/or *NRAS* mutations, compared with 9.9 months in their presence. Based on these data, Döhner et al. [11] developed a four‐gene signature (molecular prognostic risk score, mPRS) that stratified patients into three risk groups (higher benefit: not classified as intermediate‐benefit or lower‐benefit, intermediate‐benefit: *FLT3‐*ITD, *NRAS*, or *KRAS* mutation, and lower benefit: *TP53* mutation). It is now used to risk‐stratify clinical outcomes in patients treated with AZA + VEN. This four‐gene signature was further validated by Bataller et al. [12] in a cohort of 159 older (> 60 years) patients treated at the University of Texas MD Anderson Cancer Center. *NPM1* ^mut^ is considered intermediate‐risk when co‐occurring with signaling gene (SG) mutations (i.e., *FLT3*‐ITD, *NRAS*, *KRAS*) and favorable‐risk in the absence of SG mutations. However, given the small number of patients with a co‐occurring *NPM1* and *RAS* mutations (*n* = 8) or *FLT3*‐ITD (*n* = 16) in the initial cohort used to define the four‐gene prognostic risk molecular signatures, it remains unclear whether these patients truly have worse outcome compared with those without SG mutations.

We aimed to investigate the prognostic impact of *NPM1* ^mut^ with and without SG mutations in two independent cohorts of patients aged ≥ 60 years who were treated in the first line (1L) with a hypomethylating agent with VEN (HMA + VEN), with favorable‐ or intermediate‐risk by ELN 2024, and a third cohort of patients treated with HMA + VEN in the second line (2L).

## 2 Methods

### 2.1 Data Source

This retrospective observational study used data from three different patient cohorts. Cohort 1 included AML patients enrolled on the Beat AML clinical trial (NCT03013998) who met the screening criteria for enrollment and provided consent before May 10, 2023, as well as patients included in the *Myeloid Malignancy Association on Rapid Research Outcomes Working Group* (MARROW) Consortium database. Informed consent was obtained following institutional guidelines and in accordance with the Declaration of Helsinki. Cohort 2 and Cohort 3 included patients from the US nationwide Flatiron Health research database. The Flatiron Health database contains structured and unstructured data curated via technology‐enabled abstraction from approximately 280 cancer clinics and 800 unique sites of care [13, 14]. Patients who were recorded as receiving treatment with HMA + VEN as both their 1L and 2L treatments were excluded from Cohort 3, to minimize the risk of misclassifying line‐of‐therapy sequencing and to avoid potential inaccuracies in subsequent treatment capture. Informed consent was obtained following institutional guidelines and in accordance with the Declaration of Helsinki.

Patients were included if they had newly diagnosed AML with favorable‐ or intermediate‐risk by ELN 2024 (i.e., patients with a *TP53* mutation or unknown *TP53* mutation status were excluded), had a known *NPM1* mutation status, were aged ≥ 60 years at diagnosis and received treatment with HMA + VEN (Cohort 1 and 2, 1L HMA + VEN; Cohort 3, 2L HMA + VEN). Patients were excluded if they received HMA + VEN in combination with a clinical study drug, IDH or FLT3 inhibitor as 1L‐therapy, if they harbored the t(9;22)(q34;q11.2) (or were treated with a tyrosine kinase inhibitor) or if they were diagnosed with acute promyelocytic leukemia.

### 2.2 Molecular Analysis

Details of genomic analysis performed for patients enrolled on the Beat AML clinical trial have been reported previously [15]. Molecular testing was performed by next‐generation sequencing (NGS) using FoundationOne Heme (Foundation Medicine) with a limit of detection of 28 reads [16]. Molecular testing for patients included in the Marrow Consortium database was performed according to the internal guidelines of each participating academic center. The Flatiron Health research dataset captured molecular data that corresponded to a positive or negative result from specimens collected prior to the start of therapy from electronic health records, including results from fluorescence in situ hybridization (FISH), karyotyping, PCR analysis, and NGS. Patients were classified as normal karyotype only if karyotyping and/or FISH results were available and showed no abnormalities; patients without available cytogenetic data were classified as unknown.

### 2.3 Statistical Analysis

Patient characteristics are summarized using median (range) for continuous variables and frequency (percentage) for categorial variables. Student's *t*‐test or Wilcoxon rank sum test and Chi‐square or Fisher's exact test were used to compare continuous or categorial variables, respectively. Level of significance was defined as a *p* < 0.05. Birth year data for elderly patients in the Flatiron database were subject to a standard algorithmic transformation to mitigate their higher risk of patient re‐identification. Only birth year was captured and converted to the default date of December 31 (i.e., December 31, year of birth).

Overall survival (OS) was estimated using the Kaplan–Meier method from the date of trial inclusion (Cohort 1), time from 1L (Cohort 2) or 2L (Cohort 3) treatment initiation until death or last follow‐up. Group differences for censored outcomes were calculated using the log‐rank test. Univariable Cox proportional hazard models were used to describe the risk of each variable on death over time among patients with an *NPM1* mutation. Multivariable analysis was performed using the backward elimination approach starting with all variables with a *p* < 0.2 on the univariable analysis and removing the variables with the highest *p* values from the model. Statistical analyses were conducted in RStudio, Version 4.2.3 and SAS 9.4.

## 3 Results

### 3.1 Baseline Patient Characteristic Cohort 1

Cohort 1 included 322 patients treated with HMA + VEN (Table 1). Among these, 237 (74%) were classified as having favorable‐risk by ELN 2024. Patients had a median age of 73 years (range, 61–89 years), with 126 (39%) being ≥ 75 years, and 127 (39%) being female.

**TABLE 1:** Baseline characteristics of 322 patients included in Cohort 1 (*n* = 322).

Variable, *n* (%) or median (range)	*NPM1* ^mut^ (*n* = 61)	*NPM1* ^wt^ (*n* = 261)	All (*N* = 322)	*p*
Age (years)	73 (62–89)	73 (61–87)	73 (61–89)	0.95
Female sex	27 (45)	100 (39)	127 (39)	0.42
ELN 2024				
Favorable	32 (52)	205 (78)	237 (74)	**< 0.001**
Intermediate	29 (48)	56 (22)	85 (26)	
RAS/MAPK mutations				
*NRAS*	12 (20)	31 (12)	43 (13)	0.11
*KRAS*	6 (10)	17 (7)	23 (7)	0.37
*PTPN11*	11 (18)	22 (8)	33 (10)	**0.03**
*CBL*	4 (7)	20 (7)	24 (7)	0.76
*NF1* ^a^, ^b^	0 (0)	10 (7)	10 (5)	0.09
≥ 1 mutation	24 (39)	81 (31)	105 (33)	0.21
Tyrosine kinase receptor				
*FLT3*‐ITD	27 (44)	25 (10)	52 (16)	**< 0.001**
*FLT3*‐TKD	7 (11)	12 (5)	19 (6)	**0.04**
*KIT*	2 (3)	7 (3)	9 (3)	0.68
≥ 1 mutation	24 (39)	31 (12)	55 (17)	**< 0.001**
JAK/STAT				
*JAK2*	2 (3)	34 (13)	36 (11)	**0.03**
*MPL*	0 (0)	9 (3)	9 (3)	0.14
*CSF3R*	1 (2)	7 (3)	8 (2)	0.62
≥ 1 mutation	3 (5)	46 (18)	49 (15)	**0.01**
Cytogenetics				< 0.001
Normal karyotype	43 (89)	77 (42)	120 (52)	
−5 or del(5q); −7; 17/abn(17p)	0 (0)	8 (4)	8 (3)	
KMT2A‐rearrangement	0 (0)	13 (8)	13 (6)	
Inversion (16) or t(8;21)	0 (0)	3 (2)	3 (1)	
Complex karyotype	3 (7)	15 (8)	18 (8)	
Inversion (3)	0 (0)	4 (2)	4 (2)	
t(6;9)	0 (0)	0 (0)	0 (0)	
Trisomy 8	1 (2)	25 (14)	26 (11)	
Other	1 (2)	36 (20)	37 (17)	
Unknown	13	80	93	

*Note:* Bolded values indicate a statistically significant *p* < 0.05.

^a^ Missing includes unknown or not documented in the database. Missing data were not considered in *p* value calculations.

^b^ Mutation frequencies are calculated among patients with known mutation status. *NF1* mutation status was known for 190 patients.

Molecular data were available for all patients, and 61 (19%) patients had AML characterized by *NPM1* ^mut^. The median age did not differ between *NPM1* ^mut^ patients and those with wild‐type *NPM1 (NPM1* ^wt^; *p* = 0.95). Patients with *NPM1* ^mut^ AML more frequently had a co‐mutation in *PTPN11* (18% vs. 8%, *p* = 0.03), *FLT3*‐ITD (44% vs. 10%, *p* < 0.001), or the *FLT3*‐tyrosine kinase domain (TKD) mutation (11% vs. 5%, *p* = 0.04) compared to *NPM1* ^wt^. *JAK2* mutation was present in only 3% of the *NPM1* ^mut^ patients compared to 13% in those with *NPM1* ^wt^ (*p* = 0.03). The prevalence of *KRAS*, *NRAS*, *CBL*, *NF1*, *KIT*, *MPL*, and *CSF3R* mutations was not different between *NPM1* ^mut^ and *NPM1* ^wt^ patients with AML.

### 3.2 Outcomes of Patients With *NPM1* ^mut^ AML Receiving HMA Plus VEN

**FIGURE 1:**
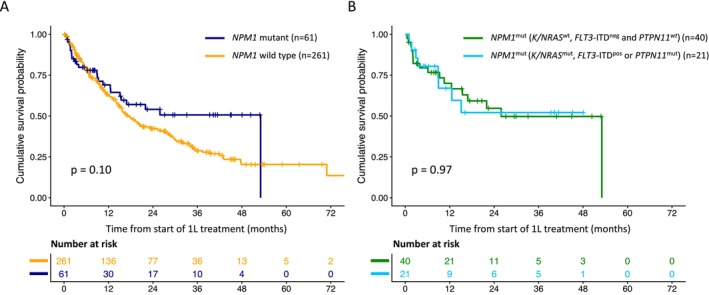
Kaplan–Meier plots of overall survival (OS) of patients stratified by *NPM1* mutation status in Cohort 1. (A) OS stratified by *NPM1* ^mut^ and *NPM1* ^wt^. (B) OS among *NPM1* ^mut^ in the presence or absence of signaling gene (SG) mutations (i.e., *KRAS*, *NRAS*, *FLT3*‐ITD, and *PTPN11*). [Color figure can be viewed at wileyonlinelibrary.com]

At a median follow‐up of 18.2 months [95% confidence interval (CI), 15.6–25.2] for the entire cohort, the median OS was 18.0 months [95% CI, 15.5–25.2] (Figure S1A). Comparing the outcomes of patients with *NPM1* ^mut^ (*n* = 61) and those with *NPM1* ^wt^ (*n* = 261), *NPM1* ^mut^ was associated with a trend toward longer OS compared to *NPM1* ^wt^ (median, 53.1 [95% CI, 12.6‐NA]) vs. 17.0 [95% CI, 14.8–21.1] months, 2‐year OS rates 54% vs. 42%; *p* = 0.10 (Figure 1A). Similar results were obtained in patients aged ≥ 75 years (*n* = 126), with a median OS of 22.0 vs. 13.9 months (*p* = 0.35) for *NPM1* ^mut^ (*n* = 24) and *NPM1* ^wt^ patients (*n* = 102).

We next evaluated the impact of specific SG co‐mutations on survival among *NPM1* ^mut^ patients. Using univariable analyses, we found that *FLT3*‐ITD (*n* = 27), *FLT3*‐TKD (*n* = 7), and mutations in *PTPN11* (*n* = 11), and *NRAS* (*n* = 12) did not significantly impact survival (Table S1). Because of the small number of patients with *NPM1* ^mut^ AML with *KRAS* (*n* = 6), *NF1* (*n* = 0), *KIT* (*n* = 2), *CBL* (*n* = 4), or *JAK*–*STAT* co‐mutations (*n* = 3), their impact on outcome could not be assessed. *FLT3*‐ITD and *PTPN11* mutations (both associated with a *p* < 0.2 in univariate analyses) were subsequently considered in multivariable modeling; however, neither emerged as an independent prognostic variable (Table S2).

The OS of *NPM1* ^mut^ patients without SG mutations (*PTPN11*, *FLT3*‐ITD, *NRAS*, or *KRAS*) (*n* = 40) did not differ from that of *NPM1* ^mut^ patients with any of those four mutations (*n* = 21) (median, 25.4 months vs. median not reached, *p* = 0.97) (Figure 1B). Moreover, the OS of patients with *NPM1* and *PTPN11* mutations (*n* = 11) was not statistically different from the OS of *NPM1* ^mut^ patients with *FLT3*‐ITD or a mutation in *NRAS* or *KRAS*, but without *PTPN11* (*n* = 28) (median, not reached vs. 25.9 months, *p* = 0.16) (Figure S2).

### 3.3 Baseline Patient Characteristics of Cohort 2

Next, we conducted similar analyses in a larger real‐world cohort (Cohort 2) of newly diagnosed AML patients. Patients had a median age of 76 years (range, 60–84 years) with 57% being aged 75 years or older, and 37% of the patients were female. Patient characteristics are shown in Table 2.

**TABLE 2:** Baseline characteristics of 816 patients included in Cohort 2 (*n* = 816).

Variable^a^, *n* (%) or median (range)	*NPM1* ^mut^ (*n* = 124)	*NPM1* ^wt^ (*n* = 692)	All (*N* = 816)	*p*
Age (years)	75 (60–84)	76 (60–84)	76 (60–84)	0.197
Female sex	60 (48)	242 (35)	302 (37)	**0.006**
ELN 2024, *n* (%)				**0.02**
Favorable	38 (43)	249 (58)	287 (55)	
Intermediate	50 (57)	183 (42)	233 (45)	
Unknown^b^	36	260	296	
RAS/MAPK mutations^c^				
*KRAS*	8 (7)	56 (8)	64 (8)	0.72
*NRAS*	16 (14)	93 (14)	109 (14)	0.98
*NF1*	9 (11)	30 (7)	39 (7)	0.25
≥ 1 mutation	28 (32)	148 (31)	176 (31)	0.90
Tyrosine kinase receptor^c^				
*FLT3*‐ITD	31 (34)	65 (16)	96 (19)	**< 0.001**
*FLT3*‐TKD	14 (17)	24 (6)	38 (8)	**0.003**
*KIT*	4 (3)	16 (2)	20 (3)	0.52
≥ 1 mutation	46 (54)	94 (24)	140 (29)	**< 0.001**
JAK/STAT signaling^c^				
*JAK2*	2 (2)	52 (8)	54 (7)	**0.015**
*MPL*	3 (3)	10 (2)	13 (2)	0.419
*CSF3R*	1 (0.1)	11 (2)	12 (2)	1.000
≥ 1 mutation	6 (6)	68 (12)	74 (11)	0.116
Cytogenetics				**< 0.001**
Normal karyotype	99 (82)	340 (51)	439 (55)	
−5 or del(5q); −7; −17/abn(17p)	4 (3)	100 (15)	104 (13)	
Trisomy 8	9 (7)	77 (11)	86 (11)	
KMT2A‐rearrangement	0 (0)	28 (4)	28 (4)	
Inversion (16) or t(8;21)	0 (0)	20 (3)	20 (3)	
Complex karyotype	1 (1)	16 (2)	17 (2)	
Inversion (3)	0 (0)	15 (2)	15 (2)	
t(6;9)	0 (0)	(0.4)	3 (0.4)	
Other	8 (7)	71 (11)	79 (10)	
Unknown	3	22	25	

*Note:* Bolded values indicate a statistically significant *p* < 0.05.

^a^ Missing includes unknown or not documented in the database. Missing data were not considered in *p* value calculations.

^b^ Patients with missing mutational status for *KRAS*, *NRAS*, or *FLT3*‐ITD, but did meet criteria for favorable or intermediate risk based on the absence of a *TP53* mutation.

^c^ Mutation status was available for the following number of patients: *KRAS* (*n* = 779), *NRAS* (*n* = 769), *NF1* (*n* = 522), ≥ 1 RAS/MAPK mutations (*n* = 565), *FLT3*‐ITD (*n* = 496), *FLT3*‐TKD (*n* = 458), *KIT* (*n* = 776), ≥ 1 tyrosine kinase receptor (*n* = 484), *JAK2* (*n* = 765), *MPL* (*n* = 676), *CSF3R* (*n* = 748), and > 1 JAK/STAT signaling mutation (*n* = 671). Mutation frequencies are calculated among patients with known mutation status.

*NPM1* ^mut^ was present in 124 (15%) patients. The median age of *NPM1* ^mut^ and *NPM1* ^wt^ patients was similar (*p* = 0.20), with a higher percentage of the *NPM1* ^mut^ patients being female (48% vs. 35%, *p* = 0.006). Patients with *NPM1* ^mut^ AML more frequently had a *FLT3*‐ITD (34% vs. 16%, *p* < 0.001) or *FLT3*‐TKD (17% vs. 6%, *p* = 0.003) co‐mutation compared to *NPM1* ^wt^ patients, whereas *JAK2* was overrepresented in *NPM1* ^wt^ patients (2% vs. 8%, *p* = 0.02). The proportions of mutations in *KRAS*, *NRAS*, *NF1*, *KIT*, *MPL* and *CSF3R* were not different between *NPM1* ^mut^ and *NPM1* ^wt^ AML patients. Mutational status for *PTPN11* was unavailable.

### 3.4 Clinical Outcomes in Cohort 2

**FIGURE 2:**
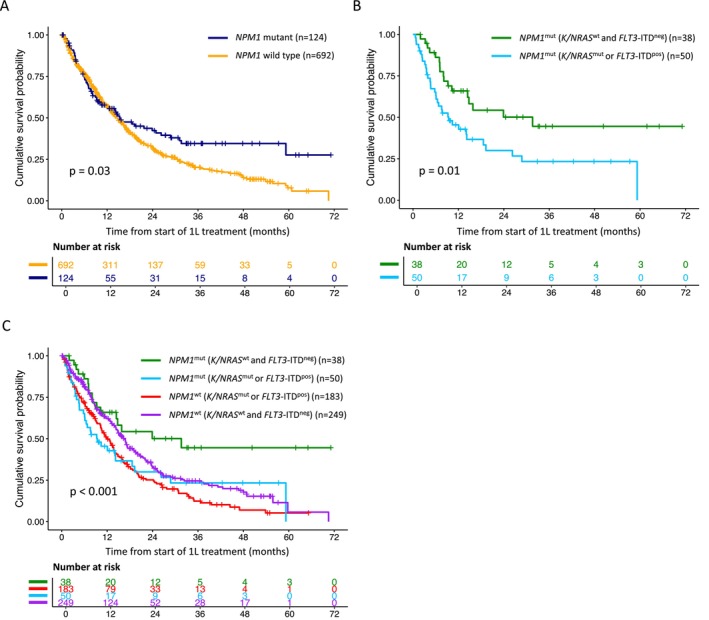
Kaplan–Meier plots of overall survival (OS) of patients stratified by *NPM1* mutation status in Cohort 2. (A) OS stratified by *NPM1* ^mut^ and *NPM1* ^wt^. (B) OS among *NPM1* ^mut^ in the presence or absence of signaling gene (SG) mutations (i.e., *KRAS*, *NRAS*, and *FLT3*‐ITD), and (C) stratified by the presence or absence of *NPM1* ^mut^ and SG^mut^. [Color figure can be viewed at wileyonlinelibrary.com]

Among all 816 patients, the median OS was 14.7 months [95% CI, 13.4–16.2] at the time of last follow‐up (median follow‐up, 10.3 months [95% CI, 9.4–11.3]) (Figure S1B). The OS of *NPM1* ^mut^ AML patients (*n* = 124) was significantly longer than OS of patients with *NPM1* ^wt^ (*n* = 692; median 15.3 vs. 14.4 months, *p* = 0.03), with 2‐year OS rates of 42.3% vs. 31.8%, respectively (Figure 2A).

As in Cohort 1, we evaluated the impact of individual SG mutations in the *NPM1* ^mut^ AML cohort of patients (*n* = 124) who received 1L HMA + VEN therapy. Using multivariable analysis, *KRAS* (*n* = 8) (HR 2.78 [95% CI, 0.95–8.10]) and *NRAS* (*n* = 16) (HR 3.04 [95% CI, 1.40–6.60]) mutations and *FLT3*‐ITD (*n* = 31) (HR 2.03 [95% CI, 1.10–3.76]) were all identified as independent unfavorable prognostic variables (Table 3, Table S3).

**TABLE 3:** Multivariable analysis in *NPM1* ^mut^ AML patients receiving HMA + VEN in the first line (*n* = 81/124).

Variable	*N* ^a^	HR	95% CI	*p*
*KRAS*	6	2.78	0.95–8.10	0.061
*NRAS*	12	3.04	1.40–6.60	**0.005**
*FLT3*‐ITD	30	2.03	1.10–3.76	**0.024**

*Note:* Bolded values indicate a statistically significant *p* < 0.05.

^a^ 81/124 had available mutation status for *KRAS*, NRAS, and *FLT3*‐ITD and were included in the multivariable analysis.

Furthermore, because Zeidner et al. [17] recently showed that *TET2* mutations were independently associated with an increased risk of death among *NPM1* ^mut^ patients in a large retrospective cohort of older patients treated with IC or lower‐intensity therapy, we evaluated the impact of *TET2* mutations in Cohort 2. Of the 124 *NPM1* ^mut^ patients, 47 (38%) had a *TET2* mutation. *TET2* mutation was significantly associated with a shorter OS, with a median of 31.6 versus 8.9 months (*p* = 0.003) (Figure S3A). The adverse prognostic significance of *TET2* mutations was also shown in the HARMONY study in patients without *FLT3*‐ITD and *DNMT3A* mutations in a cohort of intensively‐treated adults with *NPM1* ^mut^ AML [18]. Thus, we assessed the impact of *TET2* mutations in *NPM1* ^mut^ patients with *DNMT3A* ^wt^ and no *FLT3*‐ITD, and found that those who harbored *TET2* mutations (*n* = 12) had a shorter OS than those with *TET2* ^wt^ (*n* = 20) (median, 5.2 vs. 31.6 months, *p* = 0.023) (Figure S3B).

### 3.5 Outcomes of Patients With *NPM1* ^mut^ AML Stratified by the Co‐Occurrence of *KRAS* , *NRAS* Mutations, and/or *FLT3* ‐ITD

Next, we investigated whether the co‐occurrence of *KRAS*, *NRAS* mutations, and/or *FLT3*‐ITD in *NPM1* ^mut^ patients with AML impacted their survival. The presence of any of the three co‐occurring SG mutations (*n* = 50) was associated with a significantly shorter OS compared to *NPM1* ^mut^ patients with wild‐type *KRAS*, *NRAS* and without *FLT3*‐ITD (*n* = 38) (median, 9.4 vs. 31.6 months, 2‐year rates, 30.0% vs. 50.1%; *p* = 0.01) (Figure 2B). Considering the entire cohort together, patients with *NPM1* ^mut^ with a co‐occurring SG mutation (blue curve) had OS similar to that of *NPM1* ^wt^ patients with an SG mutation (red curve) (median, 11.9 vs. 9.4 months, *p* = 0.67) (Figure 2C).

### 3.6 2L HMA + VEN Cohort (Cohort 3)

We next investigated the prognostic impact of *NPM1* ^mut^ in 220 patients aged ≥ 60 years receiving HMA + VEN as 2L therapy. Patients had a median age of 73 years (range, 60–84) and 40% were female (Table 4). Thirty‐one (13%) patients had an *NPM1* ^mut^ (Table 4). Co‐mutations were largely similar between *NPM1* ^mut^ and *NPM1* ^wt^ patients, except for *FLT3*‐ITD, which was more commonly associated with *NPM1* ^mut^ (35% vs. 12%, *p* = 0.01).

**TABLE 4:** Baseline characteristics of 220 patients in Cohort 3.

Variable^a^, *n* (%) or median (range)	*NPM1* ^mut^ (*n* = 29)	*NPM1* ^wt^ (*n* = 191)	All (*N* = 220)	*p*
Age (years)	74 (61–84)	73 (60–84)	73 (60–84)	0.29
Female sex	15 (52)	72 (38)	87 (40)	0.22
ELN 2024				0.80
Favorable	15 (62)	92 (68)	107 (67)	
Intermediate	9 (38)	44 (32)	53 (33)	
Unknown^b^	5	55	60	
RAS/MAPK mutations^c^				
*KRAS*	0 (0)	16 (9)	16 (8)	0.14
*NRAS*	5 (18)	28 (15)	33 (16)	0.96
*NF1*	0 (0)	7 (6)	7 (5)	1.00
≥ 1 mutation	5 (33)	44 (34)	49 (34)	1.00
Tyrosine kinase receptor^c^				
*FLT3*‐ITD	8 (35)	15 (12)	23 (15)	**0.01**
*FLT3*‐TKD	3 (14)	4 (3)	7 (5)	0.11
*KIT*	0 (0)	4 (2)	4 (2)	1.00
≥ 1 mutation	11 (46)	23 (19)	34 (23)	**0.008**
JAK/STAT signaling^c^				
*JAK2*	0 (0)	18 (10)	18 (9)	0.14
*MPL*	2 (9)	2 (1)	4 (2)	0.07
*CSF3R*	0 (0)	7 (4)	7 (3)	0.60
≥ 1 mutation	2 (9)	26 (17)	28 (16)	0.54
First line of therapy				0.99
Intensive chemotherapy	11 (39)	69 (37)	80 (37)	
Lower intensity therapy	17 (61)	117 (63)	134 (63)	
Unknown	1	5	6	
Cytogenetics				**0.002**
Normal karyotype	27 (93)	81 (43)	108 (50)	
−5 or del(5q); −7; −17/abn(17p)	2 (7)	39 (1)	41 (19)	
KMT2A‐rearrangement	0 (0)	7 (4)	7 (3)	
Inversion (16) or t(8;21)	0 (0)	9 (5)	9 (4)	
Complex karyotype	0 (0)	3 (2)	3 (1)	
Inversion (3)	0 (0)	7 (4)	7 (3)	
Trisomy 8	0 (0)	25 (13)	25 (12)	
Other	0 (0)	16 (9)	16 (7)	
Unknown	0	4	4	

*Note:* Bolded values indicate a statistically significant *p* < 0.05.

^a^ Missing includes unknown or not documented in the database. Missing data were not considered in *p* value calculations.

^b^ Patients with missing mutational status for *KRAS*, *NRAS*, or *FLT3*‐ITD, but did meet criteria for favorable or intermediate risk based on the absence of a *TP53* mutation.

^c^ Mutation data was available for the following number of patients: *KRAS* (*n* = 209), *NRAS* (*n* = 210), *NF1* (*n* = 130), ≥ 1 RAS/MAPK mutations (*n* = 144), *FLT3*‐ITD (*n* = 151), *FLT3*‐TKD (*n* = 142), *KIT* (*n* = 212), ≥ 1 tyrosine kinase receptor (*n* = 146), *JAK2* (*n* = 204), *MPL* (*n* = 179), *CSF3R* (*n* = 202), and ≥ 1 JAK/STAT signaling mutation (*n* = 179). Mutation frequencies are calculated among patients with known mutation status.

Among the 220 patients, 39% had received IC treatment in the frontline, 61% had received lower intensity therapy, and six patients were treated with an unspecified regimen that included a clinical study drug. Eight patients had received prior treatment with midostaurin during the 1L, four patients had received ivosidenib, and one enasidenib. A summary of prior treatment regimens is shown in Table S4. Two patients received an allogeneic stem cell transplantation prior to 2L therapy.

### 3.7 Outcomes of Patients With *NPM1* ^mut^ AML Receiving HMA Plus VEN in the 2L

**FIGURE 3:**
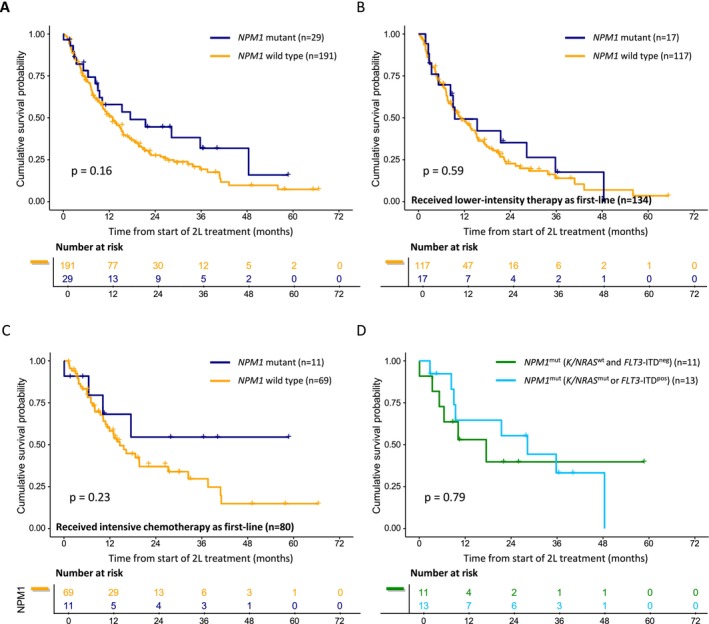
Kaplan–Meier plots of overall survival (OS) of patients stratified by *NPM1* mutation status in Cohort 3. (A) OS stratified by *NPM1* ^mut^ and *NPM1* ^wt^ among all patients treated with 2LHMA + VEN, and for patients treated with (B) lower‐intensity therapy or (C) intensive chemotherapy in the 1L. (D) OS in *NPM1* ^mut^ in the presence or absence of signaling gene (SG) mutations (i.e., *KRAS*, *NRAS*, and *FLT3*‐ITD). [Color figure can be viewed at wileyonlinelibrary.com]

At a median follow‐up of 9.7 months [95% CI, 7.9–11.3], the median OS from the start of 2L therapy was 13.0 months [95% CI, 10.3–15.6] (Figure S1C). Survival did not differ significantly between *NPM1* ^mut^ (*n* = 29) and *NPM1* ^wt^ (*n* = 191) patients (17.5 vs. 12.9 months, *p* = 0.16) (Figure 3A). When we further evaluated outcomes stratified by their 1L therapy, *NPM1* ^mut^ (*n* = 17) was not associated with OS difference compared to *NPM1* ^wt^ (*n* = 117) (median, 9.4 vs. 10.9 months; *p* = 0.59) in patients who received lower‐intensity therapy as their 1L‐therapy (*n* = 134) (Figure 3B). Although numbers were small, the OS of *NPM1* ^mut^ patients who had received IC in the 1L was slightly longer (median, 14.6 months for *NPM1* ^mut^ (*n* = 11) vs. 10.2 months for *NPM1* ^wt^ (*n* = 69), 3‐year rates, 54.5% vs. 29.7%; *p* = 0.23) (Figure 3C).

Within the *NPM1* ^mut^ cohort, univariable analysis did not identify any individual SG mutations associated with outcome (Table S5). Analysis was limited by the small sample size; only one patient had a *KRAS* mutation, and one had a mutation in the *NF1* gene. OS of *NPM1* ^mut^ cases did not differ based on the presence or absence of *NRAS/KRAS* ^mut^ and/or *FLT3*‐ITD^pos^ (*p* = 0.79) (Figure 3D).

## 4 Discussion

We describe one of the largest cohorts of older patients with newly diagnosed *NPM1* ^mut^ AML. The primary objective of this analysis was to assess the prognostic impact of co‐occurring SG mutations in older patients with *NPM1* ^mut^ AML treated with HMA + VEN. Consistent with prior studies, *NPM1* was frequently co‐mutated with *FLT3*‐ITD and *FLT3*‐TKD, and rarely with *JAK2* [19, 20]. Overall, *NPM1* ^mut^ was associated with improved OS, but the benefit was abrogated by the presence of SG mutations (*FLT3*‐ITD, *KRAS*, and *NRAS*), which is consistent with prior studies as well as ELN 2024 risk classification.

Our study included two separate cohorts of HMA + VEN in the frontline setting; Cohort 1 included patients treated in academic centers, whereas Cohort 2 included patients largely treated in a more real‐world community setting. The median OS for *NPM1* ^mut^ was 53.1 and 15.3 months compared to 17.0 and 14.4 months for *NPM1* ^wt^ in Cohorts 1 and 2, respectively. For *NPM1* ^mut^ patients with SG mutations, in Cohort 1, the OS was not statistically different from *NPM1* ^mut^ without SG mutations, whereas in Cohort 2, the median OS was 9.4 compared to 31.6 months without SG mutations. Although relapse data were not available for Cohort 1, relapse rates may have differed between patients with and without SG mutations despite similar OS outcomes, and access to salvage therapies at academic centers may have mitigated potential differences in survival outcomes.

These findings align with the four‐gene molecular risk signature, showing that although patients with *NPM1* ^mut^ AML appear to be sensitive to VEN‐based treatment, favorable clinical outcomes are largely restricted to cases lacking activated SG co‐mutations [9, 10, 11]. Similarly, the recently developed PRISM score, derived from a large multicenter cohort of 2273 newly diagnosed AML patients, confirmed the negative prognostic impact of *KRAS* mutations and *FLT3*‐ITD, but not *NRAS* mutations, irrespective of *NPM1* mutation status [21]. While our analysis was limited by the sample size of Cohort 1, and missing *PTPN11* mutational data in Cohort 2, prior studies, including the PRISM score, also suggested that *PTPN11* co‐mutations confer adverse outcomes in *NPM1* ^mut^ AML [22, 23, 24]. *PTPN11* mutations are relatively rare among all patients with AML (~8%) (24), but approximately 15%–20% of the *NPM1* ^mut^ AML patients have a co‐mutation in *PTPN11* [20, 25, 26]. Although *PTPN11* mutation status is not incorporated in the ELN 2024, and patients with *PTPN11* mutations are therefore considered “favorable‐risk,” their prognostic impact warrants further investigation and they should not be presumed to confer favorable‐risk.

Moreover, studies have also shown that *TET2* mutation may be associated with worse outcomes in patients with *NPM1* ^mut^ [17]. In cohort 2, we confirmed worse outcomes associated with *TET2* among those with an *NPM1* mutation. Due to the small number of patients in cohort 1 (*n* = 2), we were unable to assess the impact of *TET2* in this cohort. Our data suggest that *TET2* mutations may be an additional adverse‐risk factor when identified in combination with *NPM1* ^mut^ in older patients receiving AZA + VEN frontline therapy.

Next, we investigated the effect on outcome of *NPM1* ^mut^ on outcome in patients receiving HMA + VEN in the 2L. We found that *NPM1* ^mut^ was not prognostic overall. However, in patients who received IC as frontline therapy, long‐term survival appeared higher in *NPM1* ^mut^ patients though this was not statistically significant, and the sample size was small. We also found that the presence or absence of SG mutations was not prognostic in *NPM1* ^mut^ AML patients treated with HMA + VEN in the 2L setting.

The observation that SG mutations in *NPM1* ^mut^ are associated with shorter OS duration in patients treated with frontline HMA + VEN, due to either primary resistance or relapse due to acquisition or enrichment of clones with activated kinase signaling mutations [27, 28], underscores the need for new treatment options for this subset of patients. Recently, two menin inhibitors, revumenib and ziftomenib, have been FDA approved for patients with relapsed/refractory *NPM1* ^mut^ AML [29, 30]. Menin‐inhibitors target the interaction between menin and KMT2A fusion proteins or mutant NPM1, leading to downregulation of HOX/MEIS, thereby reducing leukemic persistence and promoting differentiation [31, 32]. Although limited by small numbers, both the AUGMENT‐101 and the KOMET‐001 showed activity of menin inhibitors across *NPM1* ^mut^ patients with co‐signaling gene mutations (e.g., in *KRAS/NRAS*, *FLT3*‐ITD).

The Beat AML substudy evaluated the triplet combination of AZA + VEN + revumenib for newly diagnosed older AML patients with *NPM1* ^mut^ or *KMT2A*‐rearrangement and demonstrated encouraging clinical outcomes with overall response rates and complete remission rates of 88% and 67%, respectively [33]. Further, in *NPM1* ^mut^ AML patients with SG mutations, the combination of AZA + VEN + revumenib demonstrated a composite remission rate of 77% and median OS of 15.5 months suggesting that the addition of revumenib may abrogate the poor outcomes associated with SG mutations [33]. Ziftomenib and bleximenib have also demonstrated encouraging clinical activity in combination with AZA + VEN in this patient population [34, 35]. These findings have led to three ongoing randomized phase 3 studies (KO‐MEN‐017 [NCT07007312], EVOLVE‐2: HOVON [NCT06652438], cAMeLot‐2 [NCT06852222]) investigating AZA + VEN+ menin inhibitor or placebo for newly diagnosed older/unfit AML patients with *NPM1* ^mut^. Our data corroborate prior findings that SG co‐mutations lead to worse outcomes in patients with *NPM1* ^mut^. Further data from these ongoing randomized phase 3 studies is necessary to determine whether the addition of menin inhibitors to AZA + VEN improves clinical outcomes in patients with *NPM1* ^mut^ with SG mutations.

Combinational therapies incorporating FLT3 inhibitors are also under investigation and may be effective in this higher‐risk subset. A phase 1/2 study of gilteritinib, VEN, and AZA in relapsed/refractory (*n* = 22) and newly diagnosed AML (*n* = 30) patients unfit for IC demonstrated high remission and measurable residual disease negativity rates; approximately 40% of patients harbored both an *NPM1* mutation and *FLT3*‐ITD [36]. Additional ongoing studies evaluating FLT3 inhibitor‐based triplet regimens include the phase 1/2 VICEROY trial (NCT05520567) in newly diagnosed AML with *FLT3*‐ITD or *FLT3*‐TKD, MyeloMATCH MM10A‐EA02 (NCT05564390), and a phase 1/2 trial with ASTX727 (decitabine/cedazuridine), VEN, and gilteritinib (NCT05010122).

Our study is limited by its retrospective nature and inclusion of real‐world patient data captured from electronic health records. Documentation can be incomplete, treatment sequences can be misclassified, and certain practice types or geographic regions may be overrepresented (e.g., community practices are more likely to provide data to the Flatiron database). Further, we did not have information regarding overall response rates to HMA + VEN and thus our data are only applicable to OS estimates, nor did we have data regarding subsequent therapies in Cohort 1.

In conclusion, we demonstrated that SG mutations negate the favorable prognostic impact of *NPM1* ^mut^ in patients ≥ 60 years with newly diagnosed AML treated with HMA + VEN in the 1L setting. Given the poor outcomes in this subgroup, prospective clinical trials are needed in these patients with *NPM1* ^mut^ and SG mutations. Menin inhibitors are being investigated in combination with AZA + VEN in three ongoing randomized phase 3 trials for newly diagnosed older/unfit AML patients with *NPM1* ^mut^, whereas FLT3 inhibitors are under investigation in *FLT3*‐ITD‐positive and *FLT3*‐TKD‐mutated AML, including subsets with co‐occurring *NPM1* mutations. It is imperative to assess clinical outcomes of *NPM1* ^mut^ AML patients with and without SG mutations as separate subgroups in clinical trials.

## Author Contributions

F.W.H., J.F.Z., G.T., D.N., and Y.F.M. analyzed the data. K.M., Y.H., A.L., R.L.W., R.T.S., E.T., E.M.S., T.L.L., M.R.B., V.H.D., W.G.B., M.L.A., W.S., O.O., R.L.O., C.C.S., G.J.S., E.K.C., O.C., C.M., M.H., K.S., C.F., J.G.‐L., B.K.R., S.V.H., N.A.H., T.C., M.M., M.S., S.G.M., L.R., B.J.D., R.L.L., A.S.M., A.O.Y., U.M.B., A.S.M., A.‐K.E., J.C.B., and Y.F.M. collected and assembled data, and/or were involved in patient care. F.W.H., J.F.Z., K.M., and Y.F.M. wrote the paper. All authors reviewed the manuscript and approved the final version.

## Funding

The study was sponsored by Blood Cancer United. Funding for the trial was made possible by the Harry T. Mangurian Foundation, many other donors, and the sites that enabled resources for rapid turnaround for cytogenetic analysis and other monitoring requirements for patients. Sub‐studies in this trial were supported by pharmaceutical sponsors. We thank the pharmaceutical sponsors who paid the cost of performing the specific sub‐studies with their investigational drugs.

## Ethics Statement

The study was approved by a central institutional review board (IRB) and MARROW consortium‐associated IRBs. The study was conducted in accordance with the Declaration of Helsinki and the International Council for Harmonization of Good Clinical Practice guidelines.

## Consent

All patients provided written informed consent.

## Conflicts of Interest

E.T. has participated in advisory boards and/or consulting for Abbvie, Astellas, Daiichi‐Sankyo, Servier, and Rigel. He has received research funding from Prelude Therapeutics, Schrodinger, Incyte, and Astra‐Zeneca. E.M.S. has served on the advisory boards of Astellas Pharma, AbbVie, Genentech, Daiichi Sankyo, Novartis, Amgen, Seattle Genetics, Syros Pharmaceuticals, Syndax Pharmaceuticals, Agios Pharmaceuticals, and Celgene. He is an equity holder in Auron Therapeutics. T.L.L. has received research funding from Bio‐Path Holdings, Astellas Pharma, Celyad, Aptevo Therapeutics, Cleave Biosciences, Ciclomed, Jazz Pharmaceuticals, and Kura Oncology and serves on the advisory board of Servier, Syndax, and Daiichi Sankyo. M.R.B. has institutional funding from AbbVie, Ascentage, Astellas, Gilead, Kura, Sumitomo, and Takeda. W.G.B. has served on advisory boards of AbbVie and Syndax and has research funding from ImmuneOnc, Meryx, and Nkarta. O.O. has served on the advisory boards of Servier, Rigel, ABBVIE, Incyte, and data safety monitoring board for Threadwell therapeutics. J.F.Z. has received honoraria from consulting and/or advisory fees from AbbVie, Astellas, AstraZeneca, Crossbow Therapeutics, Daiichi Sankyo, Foghorn, Genentech, Genmab, Geron, Ipsen, Jazz, Johnson & Johnson, Kura Oncology, Neogenomics, Novartis, Relmada Therapeutics, Rigel, Sellas, Servier, Shattuck Labs, Sumitomo Pharma, and Syndax; has received research funding from AbbVie, Akesobio, Aptevo, Arog, Ascentage, Auron, Daiichi Sankyo, Faron, Jazz, Kura Oncology, Lin Biosciences, Loxo, Merck, Novartis, Sellas, Shattuck Labs, Stemline, Sumitomo Pharma, and Zentalis. R.L.O. has received research funds for Cellectis and consulted for Syndax. C.C.S. has received research funds from Abbvie, BMS, Erasca, Revolution Medicines and Zentalis and served on advisory boards for Abbvie, Genentech and Astellas. G.J.S. has commercial interests in BMS, Amgen, and J&J. He has received fees from AbbVie, Agios, Amgen, Astellas, BMS, Incyte, Janssen, Jazz, Karyopharm, Kite, Pharmacyclics, Sanofi/Genzyme, and Stemline. He has received research funds from AbbVie, Actinium, Actuate, Arog, Astellas, AltruBio, AVM Bio, BMS/Celgene, Celator, Constellation, Daiichi‐Sankyo, Deciphera, Delta‐Fly, Forma, FujiFilm, Gamida, Genentech‐Roche, Glycomimetics, Geron, Incyte, Karyopharm, Kiadis, Kite/Gilead, Kura, Marker, Mateon, Onconova, Pfizer, PrECOG, Regimmune, Samus, Sangamo, Sellas, Stemline, Syros, Takeda, Tolero, Trovagene, Agios, Amgen, Jazz, Orca, Ono‐UK, and Novartis. M.S. has been a consultant for Eilean Therapeutics. B.J.D. has the following conflicts of interest: Cepheid, Labcorp, Nemucore Medical Innovations, Novartis, RUNX1 Research Program; SAB & Stock: Aptose Biosciences, Blueprint Medicines, Enliven Therapeutics, Iterion Therapeutics, GRAIL, Recludix Pharma; Board of Directors & Stock: Amgen, Vincerx Pharma; Board of Directors: Burroughs Wellcome Fund, CureOne (inactive); Joint Steering Committee: Beat AML Blood Cancer United; Advisory Committee: Multicancer Early Detection Consortium; Founder: VB Therapeutics; Sponsored Research Agreement: Astra‐Zeneca, DELiver Therapeutics, Immunoforge, Terns, Enliven Therapeutics (inactive), Recludix Pharma (inactive); Clinical Trial Funding: Novartis, Astra‐Zeneca; Royalties from Patent 6958335 (Novartis exclusive license) and OHSU and Dana‐Farber Cancer Institute (one Merck exclusive license, one CytoImage Inc. exclusive license, one DELiver Therapeutics nonexclusive license, and one Sun Pharma Advanced Research Company nonexclusive license); US Patents 4326534, 6958335, 7416873, 7592142, 10473667, 10664967, and 11049247. R.L.L. is on the supervisory board of Qiagen and is a scientific advisor to Imago, Mission Bio, Syndax. Zentalis, Ajax, Bakx, Auron, Prelude, C4 Therapeutics and Isoplexis for which he receives equity support. B.K.R. serves on the advisory boards for Astellas, BMS, and Syndax. R.L.L. receives research support from Ajax and Abbvie and has consulted for Incyte, Janssen, Morphosys, and Novartis. He has received honoraria from Astra Zeneca and Kura for invited lectures and from Gilead for grant reviews. U.M.B. has been a consultant for Genentech, Daiichi Sankyo, Takeda, Pfizer, AbbVie/Genentech, and Novartis. A.S.M. has served on the advisory boards of AbbVie/Genentech, Novartis, Ryvu Therapeutics, Rigel Therapeutics, Treadwell Therapeutics, and Foghorn Therapeutics. J.C.B. is a current equity holder in Lomond Therapeutics Inc. (a publicly traded company) and Eilean Therapeutics. J.C.B. holds membership on the Board of Directors or advisory committees of Lomond, Newave, Eilean, and Kartos, and Orange Grove. Y.F.M. has received honoraria/consulting fees from BMS, Kura Oncology, BluePrint Medicines, Geron, OncLive and MD Education, VJHemOnc, and Medscape Live. Y.F.M. participated in advisory boards and received honoraria from Sierra Oncology, Stemline Therapeutics, Blueprint Medicines, Morphosys, Taiho Oncology, SOBI, Rigel Pharmaceuticals, Geron, Cogent Biosciences, and Novartis. Y.F.M. received travel reimbursement from Blueprint Medicines, MD Education, and Morphosys. None of these relationships were related to this work. M.L.A. participated in advisory board of Syndax Pharmaceuticals. The other authors declare no conflicts of interest.

## Supporting information


**Table S1:** Univariate analysis of overall survival at last follow‐up for full Cohort 1 (*n* = 322) and *NPM1*‐mutated (*n* = 61).
**Table S2:** Multivariate analysis in Cohort 1.
**Table S3:** Univariate analysis of overall survival at last follow‐up for full Cohort 2 (*n* = 816) and *NPM1*‐mutated (*n* = 124).
**Table S4:** First line treatment regimens of patients in Cohort 3 receiving HMA + VEN in the second line (*n* = 220).
**Table S5:** Univariate analysis of overall survival at last follow‐up for full Cohort 3 (*n* = 220) and *NPM1*‐mutated (*n* = 29).
**Figure S1:** Overall survival of all patients included in (A) Cohort 1, (B) Cohort 2, and (C) Cohort 3.
**Figure S2:** Kaplan–Meier curves depicting overall survival of patients with *NPM1* ^mut^ AML who also harbored mutations in the *PTPN11* gene versus overall survival of *NPM1* ^mut^ patients with wild‐type *PTPN11* who harbored *FLT3*‐ITD, *KRAS* and/or *NRAS* mutations.
**Figure S3:** Overall survival of patients with *NPM1* ^mut^ AML (A) with and those without *TET2* mutations. (B) Overall survival of patients with *NPM1* ^mut^ AML, no *FLT3*‐ITD, and wild‐ type *DNMT3A* who harbored *TET2* mutations versus those who did not.

## Data Availability

The data that support the findings of this study are available from the corresponding author upon reasonable request.
