# Supplementary material for: Signaling Mutations Negate the Favorable Impact of NPM1 Mutations in Older Patients With Newly Diagnosed Acute Myeloid Leukemia Treated With VEN/HMA
Source: Am J Hematol. 2026 Jul 2;101(9):2246–57. doi: 10.1002/ajh.70419 (PMC13428408; doi:10.1002/ajh.70419)
Supplement: Supplementary file 1 — Table S1: Univariate analysis of overall survival at last follow‐up for full Cohort 1 (n = 322) and NPM1‐mutated (n = 61). Table S2: Multivariate analysis in Cohort 1. Table S3: Univariate analysis of overall survival at last follow‐up for full Cohort 2 (n = 816) and NPM1‐mutated (n = 124). Table S4: First line treatment regimens of patients in Cohort 3 receiving HMA + VEN in the second line (n = 220). Table S5: Univariate analysis of overall survival at last follow‐up for full Cohort 3 (n = 220) and NPM1‐mutated (n = 29). Figure S1: Overall survival of all patients included in (A) Cohort 1, (B) Cohort 2, and (C) Cohort 3. Figure S2: Kaplan–Meier curves depicting overall survival of patients with NPM1 mut AML who also harbored mutations in the PTPN11 gene versus overall survival of NPM1 mut patients with wild‐type PTPN11 who harbored FLT3‐ITD, KRAS and/or NRAS mutations. Figure S3: Overall survival of patients with NPM1 mut AML (A) with and those without TET2 mutations. (B) Overall survival of patients with NPM1 mut AML, no FLT3‐ITD, and wild‐ type DNMT3A who harbored TET2 mutations versus those who did not. [file AJH-101-2246-s001.docx]

**Supplemental Material**

**Supplemental Table S1.** Univariate analysis of overall survival at last follow-up for full Cohort 1 (n=322) and *NPM1*-mutated (n=61)

|  | **Full cohort (n=322)** | | | ***NPM1*-mutated (n=61)** | | |
| --- | --- | --- | --- | --- | --- | --- |
| **Characteristics** | **n** | **HR [95% CI]** | ***P*** | **n** | **HR [95% CI]** | ***P*** |
| *NPM1*  Wild-type  Mutated | 261  61 | 1.0  0.71 [0.46-1.08] | 0.10 |  | -  - | - |
| Sex  Male  Female | 187  127 | 1.0  1.86 [0.80-1.46] | 0.58 | 33  27 | 1.0  1.18 [0.52-2.69] | 0.68 |
| Age, years  60-74  ≥75 | 196  126 | 1.0  1.01 [0.71-1.44] | 0.95 | 37  24 | 1.0  0.79 [0.39,11.60] | 0.52 |
| *KRAS*  Wild-type  Mutated | 299  23 | 1.0  2.22 [1.32-3.71] | **0.002** | 55  6 | 1.0  Unable to calculate | NA |
| *NRAS*  Wild-type  Mutated | 279  43 | 1.0  1.24 [0.81-1.91] | 0.32 | 49  12 | 1.0  0.81 [0.27-2.38] | 0.71 |
| *RAS*  Wild-type  Mutated | 264  58 | 1.0  1.62 [1.13-2.34] | **0.008** | 46  15 | 1.0  1.49 [0.62-3.63] | 0.37 |
| *PTPN11*  Wild-type  Mutated | 289  33 | 1.0  1.29 [0.82-2.06] | 0.28 | 50  11 | 1.0  0.34 [0.08-1.44] | **0.14** |
| *CBL*  Wild-type  Mutated | 298  24 | 1.0  2.15 [1.31-3.52] | **<0.001** | 57  4 | 1.0  Unable to calculate | NA |
| *NF1*  Wild-type  Mutated | 180  10 | 1.0  0.95 [0.35-2.59] | 0.92 | 61  0 | 1.0  Unable to calculate | NA |
| *FLT3*-ITD  Absent  Present | 270  52 | 1.0  0.79 [0.52-1.21] | 0.28 | 34  27 | 1.0  0.42 [0.17-1.02] | **0.06** |
| *FLT3*-TKD  Absent  Present | 309  19 | 1.0  0.43 [0.19-0.97] | **0.04** | 64  7 | 1.0  Unable to calculate | NA |
| *KIT*  Wild-type  Mutated | 313  9 | 1.0  2.04 [0.95-4.36] | **0.07** | 59  2 | 1.0  Unable to calculate | NA |
| *JAK2*  Wild-type  Mutated | 285  36 | 1.0  1.48 [0.96-2.27] | **0.07** | 59  2 | 1.0  Unable to calculate | NA |
| *MPL*  Wild-type  Mutated | 313  9 | 1.0  1.62 [0.76-3.45] | 0.22 | 61  0 | 1.0  Unable to calculate | NA |
| *CSF3R*  Wild-type  Mutated | 285  8 | 1.0  1.10 [0.41-2.97] | 0.85 | 60  1 | 1.0  Unable to calculate | NA |

**Supplemental Table S2. Multivariate analysis in Cohort 1.**

| **Characteristic** | **HR** | **95% CI** | ***P*** |
| --- | --- | --- | --- |
| *PTPN11* | 1.05 | 0.47-2.32 | 0.91 |
| *FLT3*-ITD | 1.17 | 0.59-2.31 | 0.65 |

**Supplemental Table S3.** Univariate analysis of overall survival at last follow-up for full Cohort 2 (n=816) and *NPM1*-mutated (n=124)

|  | **Full cohort (n=816)** | | | ***NPM1*-mutated (n=124)** | | |
| --- | --- | --- | --- | --- | --- | --- |
| **Characteristics** | **n** | **HR [95% CI]** | ***P*** | **n** | **HR [95% CI]** | ***P*** |
| *NPM1*  Wild-type  Mutated | 653  124 | 1.0  0.75 [0.57-0.97] | 0.03 | 0  124 | -  - | - |
| Age, years  60-74  ≥75 | 352  464 | 1.0  1.12 [0.94-1.33] | 0.21 | 60  64 | 1.0  1.02 [0.64-1.65] | 0.93 |
| *KRAS*  Wild-type  Mutated | 715  64 | 1.0  1.44 [1.07-1.92] | 0.02 | 110  8 | 1.0  2.06 [0.82-5.2] | **0.13** |
| *NRAS*  Wild-type  Mutated | 660  109 | 1.0  1.54 [1.23-1.95] | < 0.001 | 101  16 | 1.0  2.27 [1.21-4.28] | **0.01** |
| *RAS*  Wild-type  Mutated | 622  150 | 1.0  1.51 [1.23-1.86] | < 0.001 | 96  22 | 1.0  2.07 [1.15-3.72] | **0.01** |
| *NF1*  Wild-type  Mutated | 483  39 | 1.0  2.33 [1.57-3.46] | < 0.001 | 75  9 | 1.0  1.91 [0.80-4.56] | **0.14** |
| *FLT3*-ITD  Negative  Positive | 400  96 | 1.0  1.30 [1.00-1.69] | 0.05 | 59  31 | 1.0  1.61 [0.93-2.79] | **0.12** |
| *FLT3*-TKD  Negative  Positive | 420  38 | 1.0  1.30 [0.88-1.92 | 0.19 | 68  14 | 1.0  1.43 [0.69-2.97] | 0.34 |
| *KIT*  Wild-type  Mutated | 756  20 | 1.0  1.27 [0.76-2.12] | 0.36 | 112  4 | 1.0  0.82 [0.20-3.36] | 0.78 |
| *JAK2*  Wild-type  Mutated | 711  54 | 1.0  1.18 [0.85-1.62] | 0.32 | 109  2 | 1.0  0.70 [0.10-5.04] | 0.74 |
| *MPL*  Wild-type  Mutated | 663  13 | 1.0  0.59 [0.24-1.43] | 0.24 | 96  3 | 1.0  Unable to calculate | NA |
| *CSF3R*  Wild-type  Mutated | 736  12 | 1.0  1.11 [0.58-2.15] | 0.75 | 113  1 | 1.0  Unable to calculate | NA |
| *TET2*  Wild-type  Mutated | 539  238 | 1.0  1.29 [1.07-1.55] | 0.007 | 70  47 | 1.0  2.09 [1.28-3.44] | **0.003** |

**Supplemental Table S4**. First line treatment regimens of patients in Cohort 3 receiving HMA + VEN in the second line (n=220).

| **Regimen** | **Number of patients** |
| --- | --- |
| **Intensive chemotherapy** | **80** |
| 7+3 | 40 |
| Vyxeos | 25 |
| Fludarabine, cytarabine-based | 10 |
| Intensive other | 5 |
| **Lower intensity treatment** | **134** |
| HMA | 125 |
| Azacitidine-ivosidenib | 2 |
| Azacitidine-sorafenib | 1 |
| Low-dose cytarabine | 3 |
| Enasidenib | 1 |
| Ivosidenib | 2 |
| **Other, clinical study drug** | 6 |

**Supplemental Table S5.** Univariate analysis of overall survival at last follow-up for full Cohort 3 (n=220) and *NPM1*-mutated (n=29)

|  | **All (n=220)** | | | ***NPM1*-mutated (n=29)** | | |
| --- | --- | --- | --- | --- | --- | --- |
| **Characteristics** | **n**^*^ | **HR [95% CI]** | ***P*** | **n**^*^ | **HR [95% CI]** | ***P*** |
| *NPM1*  Wild-type  Mutated | 191  29 | 1.0  0.69 [0.42-1.16] | 0.16 |  |  | - |
| Age, years  60-74  ≥75 | 129  91 | 1.0  1.51 [1.09-2.09] | **0.01** | 15  14 | 1.0  1.77 [0.64-4.89] | 0.27 |
| ELN2024  Favorable  Intermediate | 107  53 | 1.0  1.24 [0.83-1.86] | 0.30 | 15  9 | 1.0  0.56 [0.18-1.73] | 0.31 |
| *KRAS*  Wild-type  Mutated | 193  16 | 1.0  1.48 [0.82-2.69] | **0.19** | 29  0 | 1.0  Unable to calculate | NA |
| *NRAS*  Wild-type  Mutated | 177  33 | 1.0  1.43 [0.90-2.27] | 0.13 | 23  5 | 1.0  0.94 [0.26-2.34] | 0.92 |
| *RAS*  Wild-type  Mutated | 167  43 | 1.0  1.32 [0.88-1.99] | 0.19 | 23  5 | 1.0  0.94 [0.26-2.34] | 0.92 |
| *NF1*  Wild-type  Mutated | 123  7 | 1.0  1.23 [0.54 -2.93] | 0.60 | 14  0 | 1.0  Unable to calculate | NA |
| *FLT3*-ITD  Negative  Positive | 128  23 | 1.0  0.92 [0.53-1.60] | 0.76 | 15  8 | 1.0  0.90 [0.29-2.82] | 0.85 |
| *FLT3*-TKD  Negative  Positive | 135  7 | 1.0  0.55 [0.17-1.75] | 0.31 | 18  3 | 1.0  0.56 [0.07-4.38] | 0.58 |
| *KIT*  Wild-type  Mutated | 208  4 | 1.0  Unable to calculate | NA | 29  0 | 1.0  Unable to calculate | NA |
| *JAK2*  Wild-type  Mutated | 186  18 | 1.0  0.78 [0. 43-1.41] | 0.41 | 28  0 | 1.0  Unable to calculate | NA |
| *MPL*  Wild-type  Mutated | 175  4 | 1.0  Unable to calculate | NA | 20  2 | 1.0  Unable to calculate | NA |
| *CSF3R*  Wild-type  Mutated | 195  7 | 1.0  2.19 [0.96-4.99] | 0.06 | 29  0 | 1.0  Unable to calculate | NA |

^*^Due to missing data, the total number of patients does not equal n=220 or n=29 for all gene mutations.

**Supplemental Figure S1**. Overall survival of all patients included in **A)**, Cohort 1, **B)**, Cohort 2, and **C)**, Cohort 3.

**Supplemental Figure S2**. Kaplan Meier curves depicting overall survival of patients with *NPM1*^mut^ AML who also harbored mutations in the *PTPN11* gene versus overall survival of *NPM1*^mut^ patients with wild-type *PTPN11* who harbored *FLT3*-ITD, *KRAS* and/or *NRAS* mutations.

**Supplemental Figure S3**. Overall survival of patients with *NPM1*^mut^ AML **A)** with and those without *TET2* mutations. **B),** Overall survival of patients with *NPM1*^mut^ AML, no *FLT3*-ITD and wild- type *DNMT3A* who harbored *TET2* mutations versus those who did not.
